# Differential Effects of Posttranslational Modifications of CXCL8/Interleukin-8 on CXCR1 and CXCR2 Internalization and Signaling Properties

**DOI:** 10.3390/ijms19123768

**Published:** 2018-11-27

**Authors:** Alessandro Vacchini, Anneleen Mortier, Paul Proost, Massimo Locati, Mieke Metzemaekers, Elena Monica Borroni

**Affiliations:** 1 Humanitas Clinical and Research Center, via Manzoni 56, 20089 Rozzano, Milan, Italy; alessandro.vacchini@outlook.com (A.V.); Massimo.Locati@humanitasresearch.it (M.L.); Elena.Borroni@humanitasresearch.it (E.M.B.); 2 Department of Medical Biotechnologies and Translational Medicine, University of Milan, via fratelli Cervi 93, I-20090 Segrate, Italy; 3 Laboratory of Molecular Immunology, Department of Microbiology and Immunology, Rega Institute for Medical Research, KU Leuven, Herestraat 49 box 1042, B-3000 Leuven, Belgium; anneleen_mortier@hotmail.com (A.M.); mieke.metzemaekers@kuleuven.be (M.M.)

**Keywords:** chemokine, CXCL8, G protein-coupled receptor, posttranslational modifications

## Abstract

CXCL8 or interleukin (IL)-8 directs neutrophil migration and activation through interaction with CXCR1 and CXCR2 that belong to the family of G protein-coupled receptors (GPCRs). Naturally occurring posttranslational modifications of the NH_2_-terminal region of CXCL8 affect its biological activities, but the underlying molecular mechanisms are only partially understood. Here, we studied the implications of site-specific citrullination and truncation for the signaling potency of CXCL8. Native CXCL8(1-77), citrullinated [Cit5]CXCL8(1-77) and the major natural isoform CXCL8(6-77) were chemically synthesized and tested in internalization assays using human neutrophils. Citrullinated and truncated isoforms showed a moderately enhanced capacity to induce internalization of CXCR1 and CXCR2. Moreover, CXCL8-mediated activation of Gα_i_-dependent signaling through CXCR1 and CXCR2 was increased upon modification to [Cit5]CXCL8(1-77) or CXCL8(6-77). All CXCL8 variants promoted recruitment of β-arrestins 1 and 2 to CXCR1 and CXCR2. Compared to CXCL8(1-77), CXCL8(6-77) showed an enhanced potency to recruit β-arrestin 2 to both receptors, while for [Cit5]CXCL8(1-77) only the capacity to induce β-arrestin 2 recruitment to CXCR2 was increased. Both modifications had no biasing effect, i.e., did not alter the preference of CXCL8 to activate either Gα_i_-protein or β-arrestin-dependent signaling through its receptors. Our results support the concept that specific chemokine activities are fine-tuned by posttranslational modifications.

## 1. Introduction

Following entrance of an infectious agent or upon tissue injury, the generation of an appropriate and strictly controlled immune response is essential. Equipped with oxidative and non-oxidative defense strategies, neutrophil granulocytes play a central role in early immune events [1]. Their capacity to release reactive oxygen species, neutrophil extracellular traps and degradative enzymes, and their phagocytic abilities, imply that the recruitment and activation of these innate leukocytes require tight regulation. In addition to highly potent but less neutrophil-specific chemotactic factors, such as complement factor C5a and leukotriene B4, in humans, seven neutrophil-attracting chemotactic cytokines or chemokines have been identified that orchestrate directed neutrophil migration and activation time- and site-dependently [2,3,4,5]. These seven low molecular mass, chemotactic and angiogenic proteins are known as CXCL1, CXCL2, CXCL3, CXCL5, CXCL6, CXCL7 and CXCL8, with the latter being the prototype and most potent neutrophil-attracting chemokine in humans [2,6]. They all contain a conserved Glu-Leu-Arg (ELR) amino acid motif prior to two conserved Cys residues that are separated by a single random amino acid (‘X’) in their NH_2_-terminal sequence. These features imply that they are classified as ELR+ CXC chemokines that are structurally distinguished from CC, CX3C and C chemokine family members and from CXC relatives that lack the ELR motif [3,4,7]. All seven ELR+ CXC chemokines activate chemokine receptor CXCR2, although with different potency, while CXCL6 and CXCL8 also efficiently activate CXCR1 [8,9,10]. Consequently, CXCR1 and CXCR2 are considered key receptors for recruitment of human neutrophils.

CXCR1 and CXCR2 show almost 80% homology in their amino acid sequences and are conventional chemokine receptors, i.e., they are G protein-coupled receptors (GPCRs) that mainly interact with the inhibitory subtype of Gα proteins (Gα_i_) [11,12]. Thus, the classical chemokine-induced signaling through these receptors is Gα_i_ protein-dependent, mediating inhibition of adenylate cyclase and lowering the endogenous cyclic adenosine monophosphate (cAMP) levels [7,13,14,15]. Stimulation with their chemokine agonists, i.e., members of the ELR + CXC chemokine family, provokes an increase of the intracellular calcium concentration ([Ca^2+^]_i_) and ultimately results in initiation of a broad range of cellular responses, varying from cellular shape change, cytoskeleton reorganizations and cell migration, to integrin upregulation, production of reactive oxygen species, and phagocytosis [16,17,18,19]. Adequate termination of classical chemokine-induced signaling is crucial to stop the migrating cell at its final destination and to retain chemokine receptors responsive for renewed agonist stimulation. Consequently, agonist stimulation promotes internalization of chemokine receptors, resulting in reduced responsiveness after prolonged chemokine exposure [20,21,22,23,24,25,26]. Different modes of internalization can manifest, depending on the chemokine receptor involved [21]. CXCR1 and CXCR2 mainly endocytose via clathrin/dynamin-dependent mechanisms [27,28]. Here, a major role for β-arrestin adaptor proteins has been established [7,20,29].

The existence of two high-affinity CXCL8 receptors represents a first level of complexity. Evidence indicates that CXCR1 is the predominant receptor initiating phospholipase D activation and the neutrophil respiratory burst leading to reactive oxygen species production, whereas CXCR2 seems more relevant for cell migration [30,31]. Furthermore, on neutrophils, CXCL8 was found to be more potent on CXCR2 compared to CXCR1 [26]. However, on neutrophils but not on transfected HEK-293 cells or endothelial cells, CXCR1 seems to recycle to the cell membrane faster than CXCR2 [26,32]. To add even more complexity to the chemokine network, in addition to interaction with chemokine receptors, in vivo chemokine-directed leukocyte migration usually requires chemokine immobilization on glycosaminoglycans [33,34,35,36,37,38,39,40]. The latter interactions assist in generating a chemotactic gradient that navigates migration of specific leukocyte subtypes, promote chemokine oligomerization, and protect certain chemokines against processing by particular proteases [41,42,43,44]. Moreover, the exact in vitro and in vivo chemokine functioning and receptor preference are modulated by posttranslational modification, e.g., proteolysis, citrullination and nitration [41,45,46,47,48,49,50,51,52,53].

Indeed, all seven ELR+ CXC chemokines can be subjected to posttranslational processing, with CXCL8 being studied intensely in this context [41,45,47,48,50]. In addition to native CXCL8, natural CXCL8 exists in over ten differently processed isoforms, which are mainly NH_2_-terminally truncated proteins with different biological activities [45,54,55,56,57,58,59]. Also, an NH_2_-terminally elongated CXCL8 variant has been identified with two extra amino acids, probably resulting from alternative splicing of the signal peptide [60]. Consequently, when purified from cell culture supernatant of different origin, CXCL8 shows a high degree of NH_2_-terminal heterogeneity [45,54,55,56,57,58,59]. Specifically, a truncated variant lacking five NH_2_-terminal amino acids and referred to as CXCL8(6-77) is highly abundant in cell culture supernatant from stimulated leukocytes, representing almost half of the total CXCL8 yield [45]. In addition to truncated CXCL8 variants and an elongated isoform, a natural site-specifically citrullinated CXCL8 isoform, from now on called [Cit5]CXCL8(1-77), was identified [45]. [Cit5]CXCL8(1-77) derives from peptidylarginine deiminase-mediated conversion of the positively charged Arg residue at position 5 in the NH_2_-terminal sequence of CXCL8 to an uncharged citrulline (Cit) residue [45,46]. Although citrullination hardly affects the molecular mass of the chemokine (plus 1 Da), it significantly affects its biological activity. Aspects of the biological characteristics of CXCL8(6-77) and [Cit5]CXCL8(1-77) have been clarified to a certain extent [45,46,50,60,61,62], but several questions remain unanswered. Hence, we aimed to further unravel the potencies of these natural CXCL8 isoforms on the two high-affinity CXCL8 receptors CXCR1 and CXCR2.

In the present study, we aimed to detangle the complex effects of posttranslational modifications of the NH_2_-terminal region of CXCL8 on several aspects of the CXCR1- and CXCR2-signaling axes. We compared the full length, unmodified CXCL8(1-77) with the naturally occurring modified isoforms CXCL8(6-77) and [Cit5]CXCL8(1-77), not only to define the implications of specific proteolysis and citrullination of CXCL8 on Gα_i_ protein activation through CXCR1 and CXCR2, but also to investigate the consequences of these modifications on receptor coupling to β-arrestins and on receptor internalization. We found that the two modifications modulate the potency of CXCL8 on its receptors, thereby adding another level of complexity to the CXCL8-CXCR1 and CXCL8-CXCR2 signaling loops.

## 2. Results

### 2.1. Effect of Citrullination and Truncation on CXCL8-Induced CXCR1 and CXCR2 Internalization

**Figure 1:**
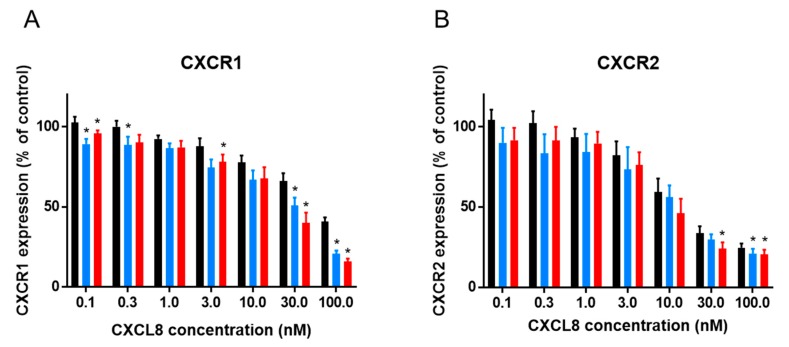
Effects of NH_2_-terminal processing on CXCL8-induced receptor internalization. Flow cytometry was used to determine CXCR1 (**A**) and CXCR2 (**B**) expression levels on freshly purified neutrophils that were stimulated with the indicated concentrations of CXCL8(1-77) (black bars), [Cit5]CXCL8(1-77) (blue bars) or CXCL8(6-77) (red bars) during 1 h. Results are represented as relative receptor expression levels (compared to buffer treated control cells) ± SEM (*n* = 6) after stimulation with the indicated concentrations of CXCL8 forms. Wilcoxon matched pairs test were performed to calculate whether stimulation with [Cit5]CXCL8(1-77) or CXCL8(6-77) induced a significantly different response compared to stimulation with native CXCL8(1-77). * *p* < 0.05 compared to CXCL8(1-77).

CXCL8 is the prototype human CXCR1 and CXCR2 agonist and mediates neutrophil chemotaxis and activation upon interaction with these receptors. However, CXCL8-mediated receptor internalization, a phenomenon thought to play a major role in correctly ceasing chemotaxis, receptor re-sensitization and turnover, also occurs. We used a flow cytometry-based approach to compare the membrane expression of CXCR1 and CXCR2 on CXCL8-stimulated neutrophils with the receptor expression on control cells stimulated with buffer. We first established that native CXCL8(1-77) significantly induced internalization of naturally expressed CXCR1 and CXCR2 on freshly purified human neutrophils from 10 nM onwards. To explore the potential effects of posttranslational modifications on the capacity of CXCL8 to induce internalization, cells were exposed to 0.1 to 100 nM CXCL8(1-77), [Cit5]CXCL8(1-77) or CXCL8(6-77). All CXCL8 isoforms clearly induced internalization of CXCR1 and CXCR2 on neutrophils in a dose-dependent manner. Moreover, CXCR1 and CXCR2 were slightly more efficiently internalized on neutrophils that were stimulated with [Cit5]CXCL8(1-77) or CXCL8(6-77) compared to cells stimulated with an equal dose of authentic CXCL8(1-77) (Figure 1). However, the effects of site-specific citrullination and truncation on CXCL8-induced receptor internalization seemed only moderate and significant differences were only observed for some doses tested.

### 2.2. Effect of Citrullination and Truncation on CXCL8-Induced Gα_i_-Dependent Signaling

**Figure 2:**
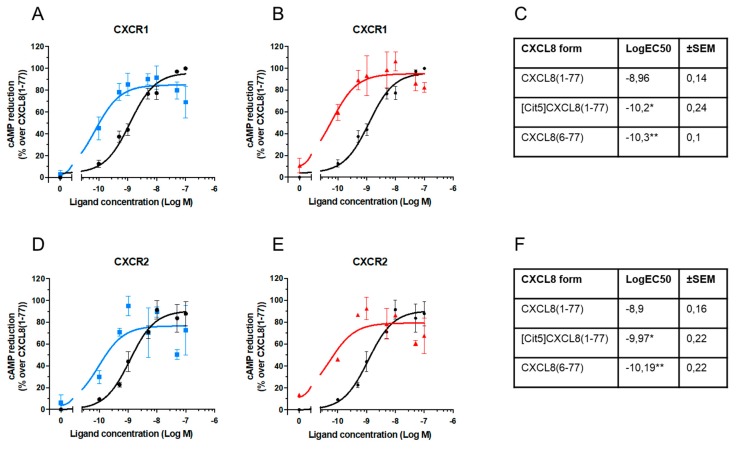
Effects of NH_2_-terminal processing on CXCL8-induced Gα_i_ protein-dependent signaling. Dose-response measurements and EC50 calculations were performed based on cAMP Alphascreen technology using HEK-293T cells transfected with CXCR1 (*n* = 3) (**A**,**B**,**C**) or CXCR2 (*n* = 2) (**D**,**E**,**F**) after stimulation with the indicated concentrations of CXCL8(1-77) ●, [Cit^5^]CXCL8(1-77) ■ or CXCL8(6-77) ▲. In dose-response plots, results are represented as percentages of activity in cAMP reduction over values obtained with authentic CXCL8(1-77) ± SEM. Mean LogEC50 ± SEM were obtained by nonlinear regression curve fitting in the cAMP assays. Results were statically compared by one-way Anova with Tukey multiple comparison. * *p* < 0.05, ** *p* < 0.01.

CXCR1 and CXCR2 are GPCRs that upon agonist stimulation mainly couple to Gα_i_ proteins, resulting in a decrease of endogenous cAMP levels. To define the capacity of CXCL8 isoforms to induce Gα_i_-signaling, CXCR1- and CXCR2-transfected HEK-293T cells were stimulated with 0.1 to 100 nM CXCL8 in the presence of forskolin. Forskolin enhances endogenous cAMP concentrations, which were measured with the Alphascreen technology [63]. Regarding CXCR1-mediated signaling, we found that the potency of [Cit5]CXCL8(1-77) and CXCL8(6-77) to activate G protein-dependent signaling was significantly enhanced, as indicated by significantly lower intracellular cAMP levels upon G protein stimulation with these isoforms as compared to native CXCL8(1-77) (Figure 2A,B). Specific EC50 values of CXCL8 forms are reported in Figure 2C. Analogously, both modified CXCL8 forms were significantly stronger inducers of G protein-dependent signaling through CXCR2 than native CXCL8(1-77) (Figure 2D–F). In general, these results showed that the potency of site-specifically truncated and citrullinated CXCL8 forms was at least ten-fold higher on both receptors.

### 2.3. Effect of Citrullination and Truncation on CXCL8-Induced β-arrestin Recruitment to CXCR1 and CXCR2

**Figure 3:**
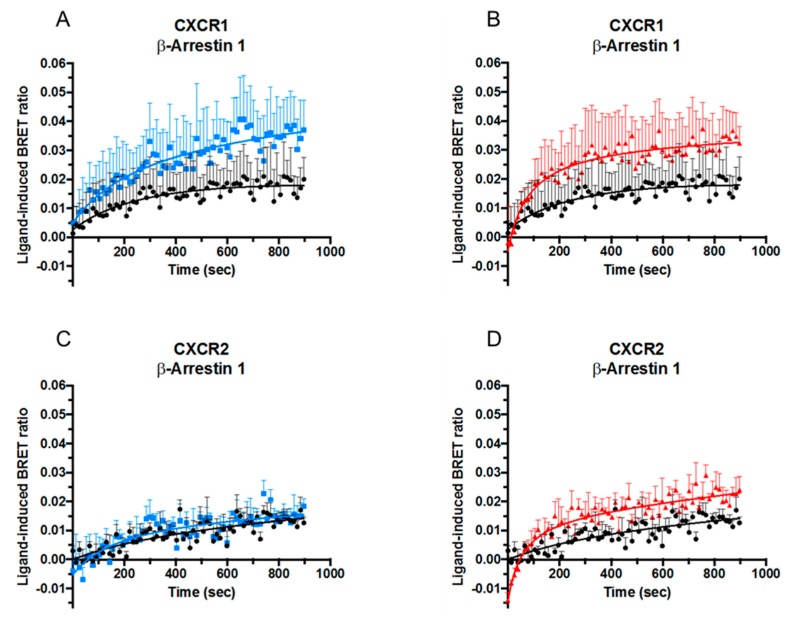
Effects of NH_2_-terminal processing on CXCL8-induced β-arrestin 1 recruitment. Kinetic BRET measurements of β-arrestin 1 recruitment to CXCR1 (*n* = 3) (**A**,**B**) and CXCR2 (*n* = 4) (**C**,**D**) in transfected HEK-293T cells after stimulation with 100 nM CXCL8(1-77) ●,[Cit^5^]CXCL8(1-77) ■ or CXCL8(6-77) ▲. Results are represented as ligand-induced BRET ratio +SEM.

**Figure 4:**
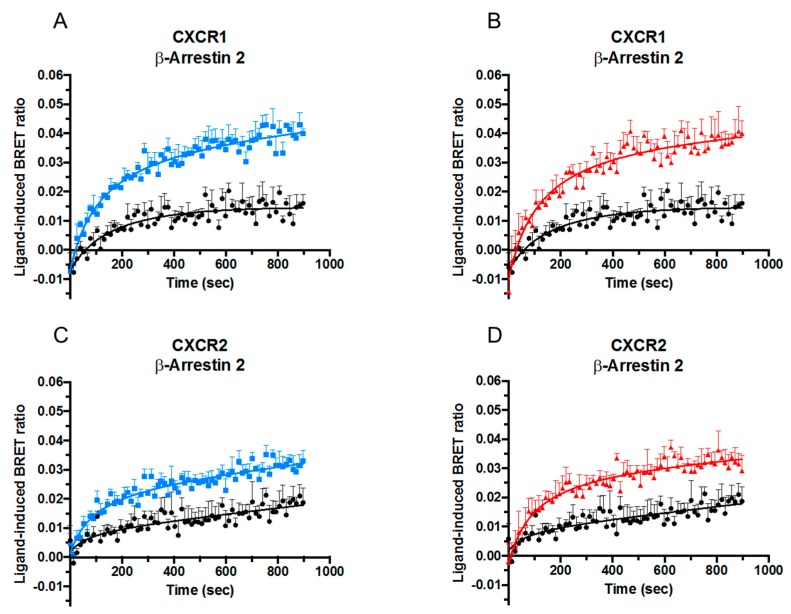
Effects of NH_2_-terminal processing on CXCL8-induced β-arrestin 2 recruitment. Kinetic BRET measurements of β-arrestin 2 recruitment to CXCR1 (*n* = 3) (**A**,**B**) and CXCR2 (*n* = 3) (**C**,**D**) in transfected HEK-293T cells after stimulation with 100 nM CXCL8(1-77) ●,[Cit^5^]CXCL8(1-77) ■ or CXCL8(6-77) ▲. Results are represented as ligand-induced BRET ratio +SEM.

**Figure 5:**
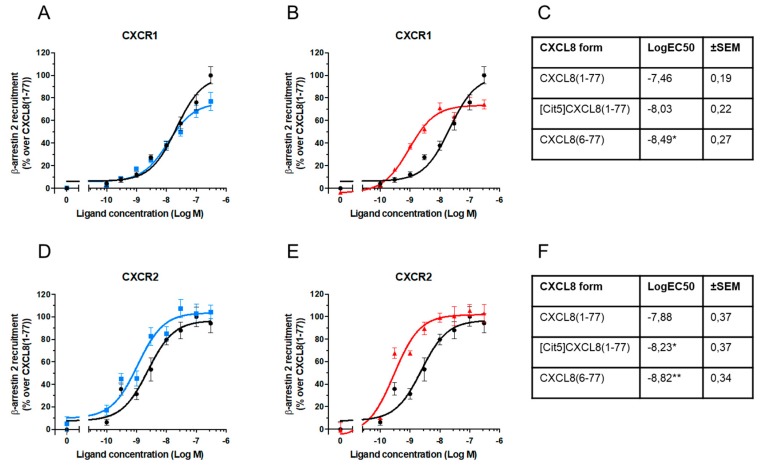
Dose-response evaluation of β-arrestin 2 recruitment induced by native and NH_2_-terminally processed CXCL8. One representative experiment is depicted showing dose-response measurements of net BRET ratio in HEK-293T cells transfected with β-arrestin 2-EYFP and CXCR1-RLuc (**A**,**B**) or CXCR2-RLuc (**C**,**D**) after stimulation with the indicated concentrations of CXCL8(1-77) ●, [Cit5]CXCL8(1-77) ■ or CXCL8(6-77) ▲. Results are represented as percentages of activity in β-arrestin 2 recruitment over values obtained with authentic CXCL8(1-77) ± SEM. Mean LogEC50 ±SEM were obtained by nonlinear regression curve fitting in the β-arrestin assays (*n* = 4) (**C,F**). Results were statically compared with one-way Anova test with Tukey multiple comparison. * *p* < 0.05, ** *p* < 0.01.

Although classical CXCR1- and CXCR2-signaling is Gα_i_ protein-dependent, ligand-stimulation also leads to initiation of signaling pathways that do not involve G proteins, among which the best known is mediated by β-arrestins. The effect of posttranslational modifications on the ability of CXCL8 to recruit β-arrestins 1 and 2 to CXCR1 and CXCR2 was therefore examined. HEK-293T cells were co-transfected with RLuc-tagged CXCR1 or CXCR2 and EYFP-tagged β-arrestin 1 or β-arrestin 2. When in close proximity, the RLuc-tag on the receptor excites the acceptor fluorophore EYFP on the β-arrestins via energy transfer resulting in light emission at 530 nm, thereby allowing investigation of β-arrestin recruitment induced by CXCL8. The obtained results were converted into the ratio of emission at 530 nm/emission at 480 nm [Bioluminescence Resonance Energy Transfer (BRET) ratio], which is related to energy transfer. CXCL8(1-77) as well as [Cit5]CXCL8(1-77) and CXCL8(6-77) induced recruitment of both β-arrestins 1 and 2 to CXCR1 (Figure 3A,B and Figure 4A,B) and CXCR2 (Figure 3C,D and Figure 4C,D). Either site-specific citrullination or loss of its five most NH_2_-terminal residues resulted in an increased potency of CXCL8 to induce β-arrestin 2 recruitment via both receptors (Figure 4) and of β-arrestin 1 recruitment through CXCR1 (Figure 3A,B). Truncation, but not citrullination, also tended to increase the potency of CXCL8 to induce β-arrestin 1 recruitment to CXCR2 (Figure 3C,D). It is worth mentioning that detection of β-arrestin 1 association seemed to occur less efficiently. As at present we cannot define whether this was a biological fact or a technical insufficiency, we focused on β-arrestin 2 recruitment. Dose-response experiments showed that citrullination did not significantly alter the potency of CXCL8 to induce β-arrestin 2 to CXCR1 (Figure 5A). In contrast, the truncated isoform CXCL8(6-77) was almost ten times more potent at inducing β-arrestin 2 recruitment to this receptor (Figure 5B,C). Regarding CXCR2-mediated signaling, [Cit5]CXCL8(1-77) as well as CXCL8(6-77) were more powerful inducers of β-arrestin 2 recruitment than native CXCL8(1-77) (Figure 5D–F).

### 2.4. No Biasing Effect of Citrullination or Truncation on CXCL8-Induced Signaling Through CXCR1 and CXCR2 

**Figure 6:**
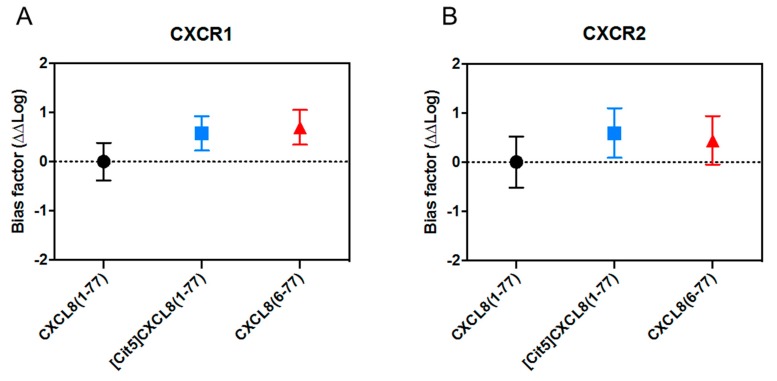
CXCL8(1-77), [Cit^5^]CXCL8(1-77) and CXCL8(6-77) are no biased ligands for CXCR1 and CXCR2. Bias plot of CXCR1 (**A**) and CXCR2 (**B**), represented as ΔΔLog for Gα_i_ signaling (cAMP reduction) against β-arrestin 2 recruitment of the different CXCL8 variants compared to natural CXCL8(1-77) ± SEM [63].

In addition to investigating the direct implications of NH_2_-terminal processing for individual signaling cascades, we wondered whether citrullination or truncation had a biasing effect on CXCL8-induced signaling. Therefore, we examined whether these modifications altered the tendency of CXCL8 to preferentially induce either Gα_i_-dependent signaling or β-arrestin recruitment. To compare these activities, we relied on the operational model of bias. This standardized model has the advantage of normalizing signals obtained with all isoforms tested, regardless of whether these are full or partial receptor agonists, and allows bias quantification without risks of system interference or observational bias that may manifest due to the experimental set up [64]. Using this approach, we calculated the transduction coefficient or log(τ/KA), with τ incorporating the ligand efficacy, receptor density and taking into account the assay system and KA being the reciprocal of the conditional ligand affinity in the assay system [65,66,67], demonstrate that neither site-specific citrullination nor removal of its five most NH_2_-terminal amino acids turned CXCL8 into a preferential inducer of Gα_i_-dependent- or β-arrestin 2 pathways for both CXCR1 and CXCR2 (Figure 6).

## 3. Discussion

From its initial discovery it has been established that natural CXCL8 exists in multiple NH_2_-terminal isoforms, with authentic CXCL8(1-77) and truncated CXCL8(6-77) likely being the most abundant [54,55,56,57,58,59]. In an inflammatory milieu, they are produced by almost any cell type, including endothelial cells [54,57], monocytes [58,59], and fibroblasts [56]. CXCL8(6-77) results from proteolytic processing of native CXCL8(1-77) by matrix metalloprotease (MMP)-8, MMP-13, MMP-14, plasmin, thrombin or cathepsin L [50,61,62,68,69,70,71], exhibits an enhanced chemotactic activity in vitro and in vivo, and is a more potent inducer of angiogenesis and Ca^2+^-signaling [45,72,73]. In the present study we demonstrated that CXCL8(6-77) more efficiently mediates Gα_i_ protein activation through CXCR1 and CXCR2 than native CXCL8(1-77). G protein-dependent signal transduction is considered the classical chemokine-induced receptor signaling pathway, enhancing the [Ca^2+^]_i_ and thereby inducing multiple cellular responses including chemotaxis, cellular shape change, cytoskeleton reorganization, integrin upregulation, production of oxygen radicals, and phagocytosis [16,17,18,19]. Thus, the initial observations that the overall biological activity of CXCL8, in general, is enhanced upon cleavage to CXCL8(6-77) [45,72,73] correlate with our data that the truncated isoform is a better initiator of Gα_i_ protein signaling through both high-affinity CXCL8 receptors.

Stimulation with high chemokine concentrations may elicit receptor internalization rather than initiating Gα_i_-dependent pathways [20,21,22,23,24,25,26], and prolonged stimulation with the chemokine ligand generally ends up in downregulation of the receptor. Indeed, it has been suggested that during leukocyte migration along a chemokine gradient, receptor internalization adequately stops the cell from migrating when the chemokine concentration is no longer increasing, thereby ceasing chemotaxis. Given the increased overall activity of CXCL8(6-77), one may speculate that the threshold of CXCL8(6-77) to induce receptor internalization is also lower compared to CXCL8(1-77). However, the results of the present study show that loss of its five most NH_2_-terminal amino acids only moderately enhances the potency of CXCL8 to induce internalization of its receptors, implying that a specific modification may have a divergent effect on different downstream pathways.

In the context of GPCR internalization, an important role has been established for β-arrestins. β-arrestins are adaptor proteins that modulate the chemokine-receptor signaling axis through interaction with phosphorylated Ser and Thr residues present in the receptor COOH-terminal tail [74,75,76,77,78,79]. However, increasing evidence suggests that also other receptor regions are essential for manifestation of β-arrestin-mediated processes, including internalization [21]. Of note, experimental evidence suggests that the effects of β-arrestins on the chemokine-receptor axis extend beyond the mere uncoupling of chemokine receptors from conventional signaling pathways and promoting receptor internalization. Specifically, in addition to their role as uncoupling molecules, β-arrestins may function as scaffolding proteins at the leading edge, where they recruit signaling molecules and play a role in actin skeleton regulation [80,81,82,83]. These features imply a complex role for β-arrestins in fine-tuning numerous migration-related intracellular pathways. The highly similar β-arrestin 1 and β-arrestin 2 have 78% identical amino acids and have been mainly compared in in vitro assays. In addition to many overlapping functions, emerging evidence suggests that they each fulfil certain unique roles, a phenomenon that likely depends on the receptor involved [84]. The only chemokine receptors studied in this context are CXCR2 [85], CXCR4 [86] and CXCR7 (also known as Atypical Chemokine Receptor 3) [87]. For CXCR2, an exclusive role for β-arrestin 2, but not β-arrestin 1, was found in upregulating β2-integrin expression, adhesion strengthening and activation of the GTPase Rap 1 [85]. In the present study, we established that CXCL8 is able to induce recruitment of both β-arrestins 1 and 2 to CXCR1 and CXCR2. Focusing on β-arrestin 2 association, we demonstrated that cleavage to CXCL8(6-77) significantly enhanced the potency of CXCL8 to mediate β-arrestin recruitment to both high-affinity CXCL8 receptors. These results may partially explain the slightly enhanced capacity of CXCL8(6-77) to induce internalization of CXCR1 and CXCR2.

The citrullinated isoform [Cit5]CXCL8(1-77) results from peptidylarginine deiminase-mediated, site-specific deimination of Arg at position 5 in the NH_2_-terminal domain of CXCL8 to Cit, and was found in cell culture supernatant from stimulated leukocytes [45]. Citrullination negatively affects the glycosaminoglycan-binding properties of CXCL8 and moderately reduces its Ca^2+^ signaling capacity through CXCR2, but slightly enhances the ability of the chemokine to upregulate integrin expression on neutrophils [45,46]. Additionally, the potency of CXCL8 to evoke ERK phosphorylation is impaired upon citrullination. Strikingly, the presence of the Cit at position 5 protects CXCL8 from potentiation by thrombin- and plasmin-mediated cleavage to CXCL8(6-77). Regarding the in vivo consequences of citrullination for the activity of CXCL8, results differ depending on the location of CXCL8 in the body. Compared to native CXCL8, the citrullinated isoform induced less neutrophil extravasation upon intraperitoneal (i.p.) injection in mice [45]. However, following intravenous injection in rabbits, citrullinated CXCL8 was a more potent neutrophil-chemoattractant than authentic CXCL8 [46]. Thus, the used experimental system and potentially the difference between either location in blood vessels or in subendothelial tissues seems of influence for the final outcome. Although concentrations of natural [Cit5]CXCL8(1-77) are probably rather low, scientific evidence suggests that protein citrullination is a more common phenomenon in an inflamed environment, such as rheumatoid arthritis [88,89]. It remains to be determined whether [Cit5]CXCL8(1-77) or other citrullinated chemokines are biomarkers for certain inflammatory diseases. Despite the fact that [Cit5]CXCL8(1-77) was originally reported to induce Ca^2+^ mobilization and ERK phosphorylation through CXCR2 on transfected HEK-293 cells less potently, we found that the citrullinated isoform is a more potent inhibitor of cAMP formation through CXCR1 and CXCR2 than native CXCL8(1-77). We also demonstrated that the potency of [Cit5]CXCL8(1-77) to induce internalization of CXCR1 and CXCR2 is moderately, enhanced compared to authentic CXCL8(1-77). Additionally, we found that its capacity to mediate recruitment of β-arrestin 2 to CXCR2, but not CXCR1, was significantly increased. Overall, these data underscore the complex effects of chemokine citrullination in general and suggest that, at least for CXCL8, different effects on specific biological activities may be observed in different experimental models.

Biased signaling, i.e., the concept that a specific receptor selectively activates one out of multiple possible signaling cascades or that a modification of the ligand leads to preference for a specific receptor, has been clearly shown for GPCRs in general and also specifically in the chemokine field. This aspect is believed to contribute to specificity in the chemokine system, as both receptor- and ligand-defined bias, as well as cell- or tissue-dependent bias, may manifest [29,90,91]. Therefore, we not only evaluated the effect of site-specific citrullination or truncation of CXCL8 in individual signaling pathways, but also examined whether these modifications turned CXCL8 into a potential biased ligand. However, although citrullination and truncation affected the potency of CXCL8 in Gα_i_ and in β-arrestin assays, these modifications did not modify the preference of CXCL8 for specific downstream signaling pathways.

To conclude, our results support the notion that NH_2_-terminal processing affects the activity of CXCL8. We demonstrated that site-specific citrullination or removal of its five most NH_2_-terminal residues significantly modulates the signaling properties of CXCL8. Both modifications increase the potency of CXCL8 to inhibit adenylyl cyclase through CXCR1 and CXCR2. The effects of truncation and citrullination on its capacity to induce internalization of CXCR1 and CXCR2 were only moderate, though significant for most doses tested. Moreover, compared to authentic CXCL8, CXCL8(6-77) showed a significantly increased ability to recruit β-arrestin 2 to its receptors. Citrullination to [Cit5]CXCL8(1-77) only significantly affected the potency of CXCL8 to induce β-arrestin 2 recruitment to CXCR2. Given the potential drastic effects of chemokine modification in general, we predict that it will be necessary to unravel the abundance and role of specific chemokine isoforms during physiological and pathophysiological conditions in humans to obtain a profound understanding of the role of chemokines in health and disease. In favor of this hypothesis, former research already demonstrated that intact CXCL8(1-77) is usually most abundant in cell culture supernatant from endothelial cells and fibroblasts, whereas stimulated leukocytes preferably process the authentic molecule into truncated and more active isoforms [55,56,57,58,59]. This may imply that chemokine processing becomes more important in inflammatory conditions, where the numbers of circulating leukocytes and release of chemokine-modifying enzymes can dramatically increase. A major future challenge will be the development of technical equipment that allows simultaneous detection and quantification of multiple chemokine isoforms in human samples.

## 4. Materials and Methods

### 4.1.Chemokines

Most conventional purification techniques, including reverse phase—high performance liquid chromatography and ion exchange chromatography, do not allow separation of individual CXCL8 isoforms from biological samples. CXCL8(1-77), [Cit5]CXCL8(1-77) and CXCL8(6-77) were therefore chemically synthesized as previously described [60,92]. Briefly, full length native CXCL8 of 77 amino acids and the natural-occurring isoforms [Cit5]CXCL8(1-77) and CXCL8(6-77) were chemically synthesized with Fmoc chemistry using an Activo P11 solid-phase peptide synthesizer (Activotec, Cambridge, UK). The synthesized chemokines were purified to homogeneity by reverse phase—high performance liquid chromatography on a Source 5-RPC column (GE Healthcare, Uppsala, Sweden) and elution of the synthesized proteins was performed with an acetonitrile gradient in 0.1% (*v/v*) trifluoroacetic acid, with 0.7% of the column effluent being directly injected into a Bruker Amazon SL electrospray–ion trap mass spectrometer (Bruker Daltonics, Bremen, Germany). Online mass spectrometry was used to select fractions containing homogeneous CXCL8 isoforms. These were pooled, evaporated and diluted in ultrapure water. The purified CXCL8 isoforms were folded into their correct configuration as described [92] and analyzed by mass spectrometry. The identity of the chemokines was also confirmed with automated NH_2_-terminal sequencing based on the principle of Edman degradation (Procise 491 cLC sequencer, Applied Biosystems, Foster City, CA, USA). Lastly, specific CXCL8 ELISAs, sulfate-polyacrylamide gel electrophoresis (SDS-PAGE) and bicinchoninic acid protein assays (Pierce, Woodland Hills, CA, USA) were conducted to examine chemokine concentrations and purity [92,93].

### 4.2. Cells

HEK-293T cells (American Type Culture Collection (ATCC), Manassas, VA, USA) used in cAMP and β-arrestin assays were grown as monolayer cultures in Dulbecco’s Modified Eagle’s Medium (DMEM; Lonza, Allendale, NJ, USA) enriched with 10% (*v/v*) fetal bovine serum (FBS; Lonza), 10 mM HEPES (Lonza) and 100 μg/mL penicillin-streptomycin (P/S; Lonza). Twenty-four hours prior to transfection, the cells were harvested using trypsin/EDTA (Lonza) and seeded in 10 cm diameter-tissue culture dishes at final concentrations of 4.5 × 10^6^ cells/mL. Human neutrophils were isolated from fresh blood after removal of mononuclear cells by centrifugation (10 min, 218 g, RT) in a density gradient (Pancoll human, 1.077 g/mL; PAN Biotech GmbH, Aidenbach, Germany). Erythrocytes were eliminated by hypotonic shock with ultrapure water (30 s).

### 4.3. Plasmids

Plasmids encoding NH_2_-terminal hemagglutinin (HA)-tagged human CXCR1 and CXCR2 were generated by PCR-amplifying CXCR1 (HA-CXCR1 forward: 5′- ATGGGCTACCCATACGACGTCCCAGACTACGCTTCAAATATTACAGATCCACAGA-3′, CXCR1 reverse: 5′- TCTGTCAGAGGTTGGAAGAGACATTGA-3′) and CXCR2 (HA-CXCR2 forward: 5′- ATGGGCTACCCATACGACGTCCCAGACTACGCTGAAGATTTTAACATGGAGAG-3′, CXCR2 reverse: 5′- TCAGTTAGAGAGTAGTGGAAGTGTGC-3′) coding sequences. A HindIII restriction site was added before the HA start codon with a second PCR reaction, in which the oligo HindIII-HA (5′- CGCTAAGCTTGACATGGGCTACCCATACGAC-3′) was used as forward primer for both coding sequences while the same reverse oligonucleotides from the first reaction were used. PCR products were cloned in pcDNA6/V5-His A (Invitrogen, Carlsbad, CA, USA) between HindIII and EcoRV restriction sites. Plasmids encoding human CXCR1 and CXCR2 flanked with HA and Renilla luciferase (Rluc) tags at the NH_2_- and COOH-terminus, respectively, were obtained by PCR-amplification of HA-tagged receptor sequences with the oligonucleotide HindIII-HA as forward primer and the oligonucleotide 5′- GGATCCCGGGCGAGGTTGGAAGAGACATTGA-3′ specific for CXCR1 and 5′- GTATCCCGGGCGAGAGTAGTGGAAGTGTGCC-3′ for CXCR2, to remove stop codons before cloning them between HindIII and SmaI restriction sites in pRLuc-N3 vectors (PerkinElmer, Norwalk, CT, USA). β-arrestins 1 and 2, fused to an ‘Enhanced yellow-green variant of the Aequorea Victoria green fluorescent protein’ (EYFP)-tag, were produced in pEYFP-N1 vectors (Clontech, Takara Bio Inc, Japan) cloning the corresponding bovine sequences between restriction sites HindIII and AgeI after PCR amplification of β-arrestin 1 with the oligonucleotides 5′- CGTGAAGCTTACCATGGGCGACAAAGGGA-3′ and 5′-CATGACCGGTGGTCTGTCGTTGAGCCGC3′ and β-arrestin 2 with primers 5′- CGTGAAGCTTACCATGGGGGAGAAACCCC-3′ and 5′- GATGACCGGTGGGCAGAACTGGTCGTCATAG-3′ after removal of an internal AgeI with the overlapping mutagenic oligonucleotides 5′-ATGTCTGACAGGTCCCTGCA-3′ and 5′ TGCAGGGACCTGTCAGACAT-3′. All plasmids were verified by Sanger sequencing.

### 4.4. Transient Co-Transfection for β-Arrestin Recruitment and cAMP Assays

For β-arrestin recruitment assays, HEK-293T cells were harvested using Trypsin/EDTA and 3 × 10^6^ cells were seeded in 10 cm diameter-tissue culture dishes twenty-four hours prior to transfection. DNA mixtures comprising 8 μg β-arrestin 1-EYFP, β-arrestin 2-EYFP or empty pcDNA3 plasmid and 2.4 μg HA-CXCR1-RLuc or HA-CXCR2-RLuc were prepared in a total volume of 1.5 ml Opti-MEM (Gibco, Life Technologies, Carlsbad, CA, USA) and 32 μg of the transfection reagent polyethylenimine (PEI; Polyscience, Eppelheim, Germany) were added, mixed and incubated at room temperature (RT) for 20 min. For cAMP experiments, HEK-293T cells were transfected according to the same protocol, using 32 μg PEI to transfect 10,5 μg plasmids obtained after cloning cDNA encoding human CXCR1 or CXCR2 into pcDNA6 vector (Invitrogen). DNA/PEI mixtures were dropwise distributed on cells kept in DMEM without antibiotics supplemented with 10% (*v/v*) FBS and incubated for 48 h. The expression of β-arrestins and chemokine receptors was verified by flow cytometry. 

### 4.5. β-arrestin Recruitment Assay

The chemokine-induced recruitment of β-arrestins to CXCR1 and CXCR2 was examined using the Bioluminescence Resonance Energy Transfer 1 (BRET1) technique [63]. Transfected cells (*vide supra*) were washed with PBS and harvested using 0.02% (*w/v*) EDTA (Lonza). The amount of protein was determined with a Bio-Rad DC protein assay kit (BioRad, Hercules, CA, USA) and cell pellets were resuspended at final concentrations of 1 mg/mL in PBS enriched with 0.1% (*w/v*) glucose to seed 80 μg of cells per well in an opaque white well/black frame 96-well plate (Perkin Elmer, Waltham, MA, USA). For kinetic BRET measurements 5 μM coelenterazine H was added and after incubation in the dark for 8 min at RT, PBS or chemokine variants at final concentrations of 100 nM were added and light emission was immediately measured at 460–500 (RLuc) and 510–550 (EYFP) sequentially for each well using a Synergy H4 Hybrid reader (Biotek, Bad Friedrichshall, Germany). To perform dose-response experiments, 80 μg of cells were seeded per well in an opaque white well/black frame 96-well plate. Different concentrations of CXCL8 forms were added and the plate was incubated at 37 °C for 15 min before the addition of 5 μM coelenterazine H and further 8 min incubation at RT, before light emission measurement. BRET ratios were calculated dividing luminescence values obtained with the EYFP filter by the values obtained using the RLuc filter, and the ligand effect on BRET was obtained subtracting BRET values obtained by cells stimulated with PBS from BRET values obtained by cells stimulated with the different CXCL8 variants.

### 4.6. cAMP Assay

To measure intracellular cAMP, the AlphaScreen cAMP assay (Perkin Elmer) was used according to the manufacturer’s instructions. Briefly, CXCR1- and CXCR2-transfected HEK-293T cells (*vide supra*) were harvested and washed with PBS prior to being resuspended at final concentrations of 10^7^ cells per ml in freshly prepared stimulation buffer composed of 1 × ‘Hanks’ Balanced Salt Solution’ (HBSS; Lonza) complemented with 0.1 % (*w/v*) BSA, 0.5 nM IBMX (Sigma-Aldrich) and 5 mM HEPES (Lonza). Per well, 10^4^ cells were plated in a 384-well white opaque plate (Perkin Elmer) with 0.2 units/μL anti-cAMP acceptor beads in 5μL volume, to which 5μL of 10 μM forskolin and serial chemokine dilutions were added. After 30 minutes incubation at RT in the dark, 15μL of lysis buffer containing 1 Unit of streptavidin-labeled donor beads and 1 Unit biotinylated cAMP were added and the plate was incubated at RT in the dark for 1 h. The luminescence of the beads was read on a Synergy H4 plate reader through a 570/100 nm filter after sample excitation with 680/30 nm filtered light.

### 4.7. Internalization Assay 

The potency of CXCL8 isoforms to induce internalization of CXCR1 and CXCR2 on neutrophils was investigated using a flow cytometry-based approach, as previously described [94]. Freshly purified human neutrophils were resuspended in RPMI1640 medium (Cambrex Corporation, East Rutherford, NJ, USA) complemented with 0.5% (*w/v*) human serum albumin (HSA; Red Cross Blood transfusion center, Leuven, Belgium) at final concentrations of 5 × 10^6^ cells per ml. Per well, 5 × 10^5^ cells were plated in a round bottom 96-well plate, which was placed at 37 °C for 5 min. Cells were stimulated with 0.1 to 100 nM CXCL8 isoforms for 1 h at 37 °C. Stimulation with solely buffer was used as a control. The plate was kept on ice for 10 min, centrifuged (5 min, 315 g, 4°C), and pellets were diluted in PBS enriched with 2% (*v/v*) fetal bovine serum (FBS; Invitrogen). Following centrifugation (5 min, 315 g, 4 °C), cells were stained with APC-labeled mouse anti-human CXCR1 and PE-labeled mouse anti-human CXCR2 antibodies (BD Pharmingen). After 30 min incubation in the dark on ice, cells were centrifuged (5 min, 315 g, 4 °C) and washed two times with PBS + 2% (*v/v*) FBS. Final pellets were resuspended in PBS enriched with 2% (*v/v*) FBS and 0.4% formaldehyde. Fluorescence intensities were determined with a BD FACSCaliburTM flow cytometer (BD Biosciences) and results were analyzed with compatible software. Relative mean receptor expression levels were calculated as a percentage as 100 × (MFI_CXCL8_) / (MFI_Buffer_), with ‘MFI_CXCL8_’ and ‘MFI_Buffer_’ being the mean fluorescence intensities (MFI) obtained after treatment with CXCL8 or buffer, respectively.

### 4.8. Bias Calculation

To determine whether post-translational modifications of CXCL8 can induce functional selectivity on CXCR1 and CXCR2, we calculated for each isoform its Transduction Coefficient (TC = log(τ/KA)). First, using Graphpad Prism we fitted the Black-Leff operational model to dose-response curves of cAMP reduction and β-arrestin 2 recruitment [63,65]. Native CXCL8(1-77) was considered as reference ligand. Therefore, we calculated for each assay the relative effectiveness of each post-translationally modified CXCL8 variant subtracting from its TC the TC of native CXCL8 (Δlog(τ/KA) = log(τ/KA)CXCL8 isoform − log(τ/KA)CXCL8(1-77)). Relative effectiveness of each chemokine isoform was then used to calculate the bias factor (ΔΔlog(τ/KA) = Δlog(τ/KA)cAMP reduction − Δlog(τ/KA)β-arrestin 2 recruitment) which reflects the ability of each post-translational modification to preferentially activate one single signaling pathway downstream of each receptor.

### 4.9. Statistical Analysis

Wilcoxon matched pairs tests were performed to evaluate whether results of paired groups in internalization assays were significantly different or not. LogEC50 values obtained in cAMP and β-arrestin assays and bias factors were statistically compared using one-way ANOVA tests with Tukey’s multiple comparison. A *p* value of 0.05 or less was considered significant.

## Abbreviations

[Ca^2+^]_i_	Intracellular calcium concentration
ELR	Glu-Leu-Arg
GPCR	G protein-coupled receptor
